# Systemic Administration of siRNA *via* cRGD-containing Peptide

**DOI:** 10.1038/srep12458

**Published:** 2015-08-24

**Authors:** Yuanyu Huang, Xiaoxia Wang, Weiyan Huang, Qiang Cheng, Shuquan Zheng, Shutao Guo, Huiqing Cao, Xing-Jie Liang, Quan Du, Zicai Liang

**Affiliations:** 1 Institute of Molecular Medicine; State Key Laboratory of Natural and Biomimetic Drugs, School of Pharmaceutical Sciences, Peking University, Beijing 100871, China; 2 Chinese Academy of Sciences Key Laboratory for Biomedical Effects of Nanomaterials and Nanosafety, National Center for Nanoscience and Technology of China, Beijing 100190, China; 3 Collaborative Innovation Center of Chemical Science and Engineering (Tianjin), Tianjin 300072, China

## Abstract

Although small interfering RNAs (siRNAs) have been demonstrated to specifically silence their target genes in disease models and clinical trials, *in vivo* siRNA delivery is still the technical bottleneck that limits their use in therapeutic applications. In this study, a bifunctional peptide named RGD10-10R was designed and tested for its ability to deliver siRNA *in vitro* and *in vivo*. Because of their electrostatic interactions with polyarginine (10R), negatively charged siRNAs were readily complexed with RGD10-10R peptides, forming spherical RGD10-10R/siRNA nanoparticles. In addition to enhancing their serum stability by preventing RNase from attacking siRNA through steric hindrance, peptide binding facilitated siRNA transfection into MDA-MB-231 cells, as demonstrated by FACS and confocal microscopy assays and by the repressed expression of target genes. When RGD10 peptide, a receptor competitor of RGD10-10R, was added to the transfection system, the cellular internalization of RGD10-10R/siRNA was significantly compromised, suggesting a mechanism of ligand/receptor interaction. Tissue distribution assays indicated that the peptide/siRNA complex preferentially accumulated in the liver and in several exocrine/endocrine glands. Furthermore, tumor-targeted delivery of siRNA was also demonstrated by *in vivo* imaging and cryosection assays. In summary, RGD10-10R might constitute a novel siRNA delivery tool that could potentially be applied in tumor treatment.

Small interfering RNAs (siRNAs) are able to silence almost any target gene after cellular delivery, providing them with great potential for gene therapy for various diseases, including cancer^1^,^2^, hepatitis B caused by HBV^3^, Ebola hemorrhagic fever (EHF)^4^, TTR-mediated amyloidosis (ATTR)^5^, and diabetic macular edema (DME)^6^, among others. However, the relatively large size and negative charge of the molecules have constituted the greatest limitations for their clinical application. Consequently, *in vivo* delivery methods have been widely explored to overcome these obstacles.

Using both the endocytotic pathway and non-endocytotic pathway (transduction)^7^,^8^, cell-penetrating peptides (CPPs) are attractive tools for the delivery of various cargos to targeted cells^9^,^10^,^11^. The cargos can associate with the CPP through either covalent chemical bonds or non-covalent interactions^12^. Polyarginine is a cationic CPP that can cross the cell membrane^13^,^14^ and has been used to decorate certain carriers to enhance their transfection efficiency^15^,^16^. Manjunath and colleagues have demonstrated that RVG-9dR, a fusing peptide that contains a RVG motif derived from rabies virus glycoprotein and nona-D-arginine residues (9dR), enabled the transvascular delivery of siRNA to the central nervous system by specifically binding to the acetylcholine receptor expressed on neuronal cells^17^. Another study further demonstrated that this peptide could mediate siRNA delivery into macrophages for anti-inflammatory treatment^18^. In this case, polyarginine was incorporated to bind siRNA through electrostatic interactions.

Arginine-glycine-aspartic acid (RGD) tripeptide is receptor-recognition motif that specifically binds to integrins α_v_β_3_ and α_v_β_5_^19^. Integrins α_v_β_3_ and α_v_β_5_ are both heterodimeric cell surface receptors that have been found to mediate adhesion between cells and the extracellular matrix (ECM) and to stimulate intracellular signaling and gene expression involved in cell growth, migration and survival^20^. Integrin α_v_β_3_ is the predominant receptor of the RGD motif and is highly expressed in tumor cells and neovascular endothelial cells^21^,^22^,^23^. The diverse applications of RGD peptides include inhibiting apoptosis, angiogenesis and tumor formation^24^; imaging for diagnostic purposes^25^,^26^; and coating surfaces for use as biomaterials to enhance drug delivery systems^27^. Although RGD-based peptides have been used to facilitate siRNA transportation *in vitro* and *in vivo*, they were only incorporated into certain carriers and simply played a role of specific recognition^28^,^29^,^30^,^31^,^32^. Such types of delivery systems are typically multicomponent, complex and difficult to develop as potential drug delivery formulations.

In this study, RGD10, a representative RGD-motif-containing peptide with an amino acid sequence of DGARYCRGDCFDG, identified by Kontermann and colleagues using phage display technology^33^, was fused with decu-L-arginine residues (10R) to form a bifunctional CPP termed RGD10-10R. RGD10 is a cyclic peptide that contains a disulfide linkage between the two cysteines. It was previously reported that the cyclic RGD peptide exhibited considerably higher affinity toward α_v_β_3_ compared with the linear RGD peptide^34^. In this delivery system, RGD10 was designed to recognize integrin(s) expressed on tumor cells and tumor-associated endothelial cells, and 10R was employed to bind siRNA through electrostatic interactions (Fig. 1a). The physicochemical properties of the RGD10-10R/siRNA complex, including siRNA retarding ability, morphology, hydrodynamic diameter, zeta potential, and RNase resistance, were characterized. Cytotoxicity, intracellular uptake and localization, and specific gene silencing were assessed *in vitro*. Furthermore, we evaluated the *in vivo* distribution and tumor-targeting profile through fluorescent imaging and cryosection observations.

## Results

### Gel retardation assay

To evaluate the loading capability of RGD10-10R, a gel retardation assay was performed using si-NC-67M, a serum-resistant siRNA. The use of this siRNA could exclude the possibility that degradation of the siRNA affected its gel shift. Two and a half microliters of siRNA (20 μM, ~50 pmol) was mixed with a given amount of the peptide and incubated at room temperature for 15–20 min to form RGD10–10R/siRNA complexes with different molar ratios. The complexes were adjusted to identical volumes with DEPC water; then, they were loaded into a 4% agarose gel and separated for 40 min at a constant voltage of 140 V. UV imaging revealed that when the molar ratio of peptide and siRNA increased from 25:1 to 200:1, increasingly less siRNAs moved in the gel from the negative pole to positive gel to form electrophoresis bands, suggesting that more siRNAs were entrapped in the complexes with increasing amounts of the peptide. Moreover, when the molar ratio reached 25:1, the majority of the siRNAs were already entrapped in the complexes (Fig. 2a, 1b-left panel). As expected, when the molar ratio increased to more than 100:1, the complexes ran in the opposite direction (from the positive pole to the negative pole) (Fig. 2b-left panel). Compared to RGD10-10R, compromised siRNA binding capacity was observed for polyarginine (10R) peptide without the RGD10 motif at the N terminal (Fig. 2b-right panel). The influence of Opti-MEM, an optimized medium used for siRNA transfection, on the stability of the complex was further assessed. The results indicated that RGD10-10R could bind siRNA and form stable complexes in this medium, as well as in DEPC water (Fig. 2c).

### RNase resistance assay

To test the hypothesis that the formation of complexes could protect siRNAs from RNase attack and therefore enhance their stability, an RNase resistance assay was performed using an unstable double-stranded small RNA termed mmu-miR-672. The results indicated that when incubated in 10% FBS at 37 °C, these small RNAs were rapidly degraded (Fig. 2d-left). In contrast, when the small RNAs were encapsulated by the peptides, full-length bands were clearly observed, even with a prolonged incubation of 12 hours. In addition, if the peptide/siRNA complexes were not treated with proteinase K, the peptides were not digested and the complexes were not destroyed, and they could not move in the gel from the negative pole to the positive pole, which further confirmed that the siRNAs were thoroughly entrapped by the peptides (Fig. 2d-right panel, the second lane). Here, RGD10-10R/siRNA with a molar ratio of 100:1 was not observed to run from the positive pole to the negative pole because no gel existed in the opposite direction, which was different from the agarose gel used in the gel retardation assay.

### Physicochemical properties of RGD10-10R/siRNA complexes

**Figure 1:**
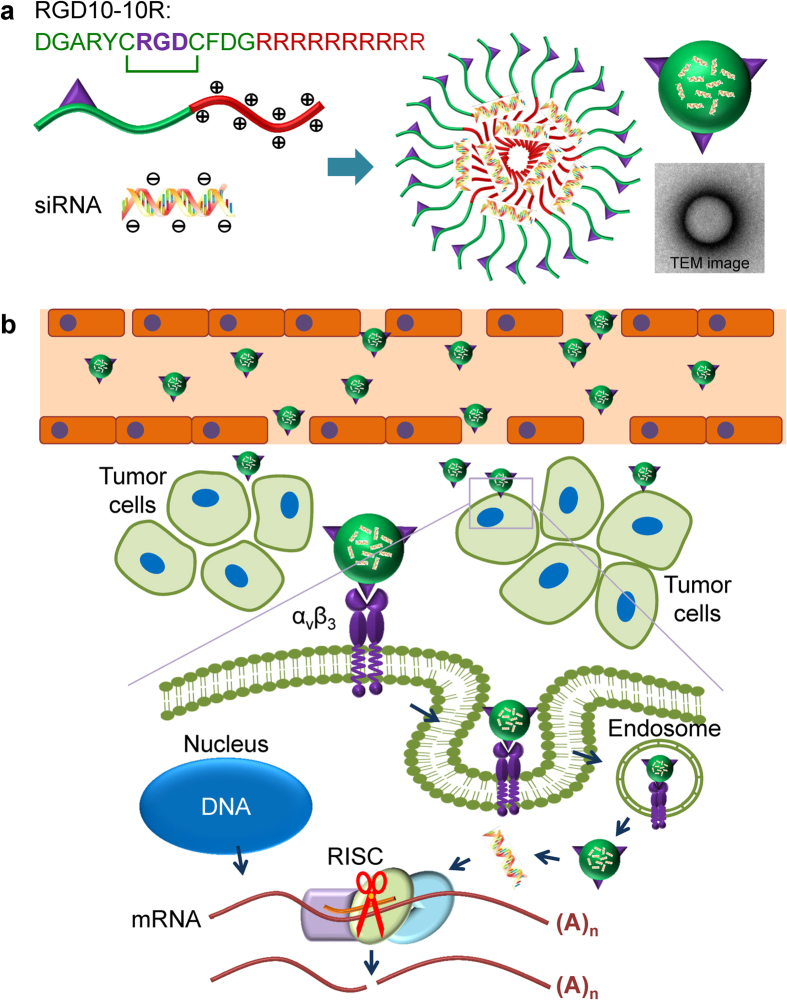
Fabrication of the RGD10-10R/siRNA complex (**a**) and tumor-targeted siRNA delivery involving ligand/receptor interactions (**b**). (**a**) RGD10-10R/siRNA complexes were prepared by directly mixing RGD10-10R peptide and siRNA at desired molar ratios followed by incubation at room temperature for 15–20 min. (**b**) siRNAs accumulated in the tumor tissue and then entered the tumor cells in a receptor (α_v_β_3_)-mediated endocytosis (RME) manner *in vitro*. After being internalized by cells, peptide/siRNA complexes escaped from the endosomes/lysosomes. Then, siRNAs were released from the complexes and loaded by RNA-induced silencing complex (RISC). Targeted messenger RNA complementary to the guide strand (antisense strand) of siRNA was selected and cleaved by argonaute protein. All parts of this figure were drawn by the author Y. H.

**Figure 2:**
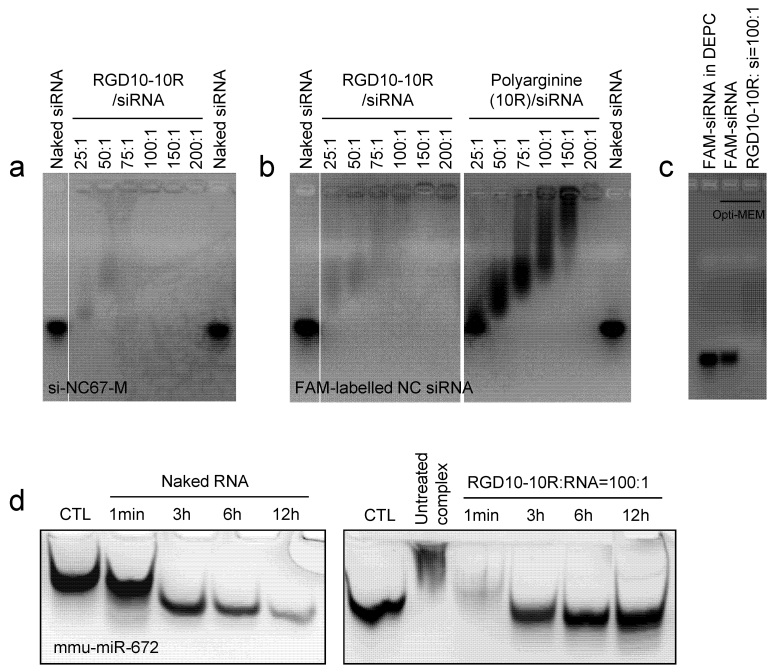
Gel retardation (**a–c**) and RNase resistance (**d**) assays of RGD10-10R/siRNA complexes. The siRNA loading capacity of RGD10-10R was analyzed using two serum-stable siRNAs: si-NC67-M (**a**) and FAM-labeled NC siRNA (b-left panel). Polyarginine (10R) was used as a control (b-right panel). The complex formed in Opti-MEM was examined to assess the siRNA binding capability of the peptide and the stability of the complexes (**c**). An RNase resistance assay was performed to evaluate the protection effect resulting from encapsulation of the peptides using an unstable small RNA termed mmu-miR-672. Both naked RNAs (d-left panel) and complexes (d-right panel) were separated in a 20% native PAGE. These data demonstrated that RGD10-10R binds siRNAs and effectively protects them from RNase degradation.

**Figure 3:**
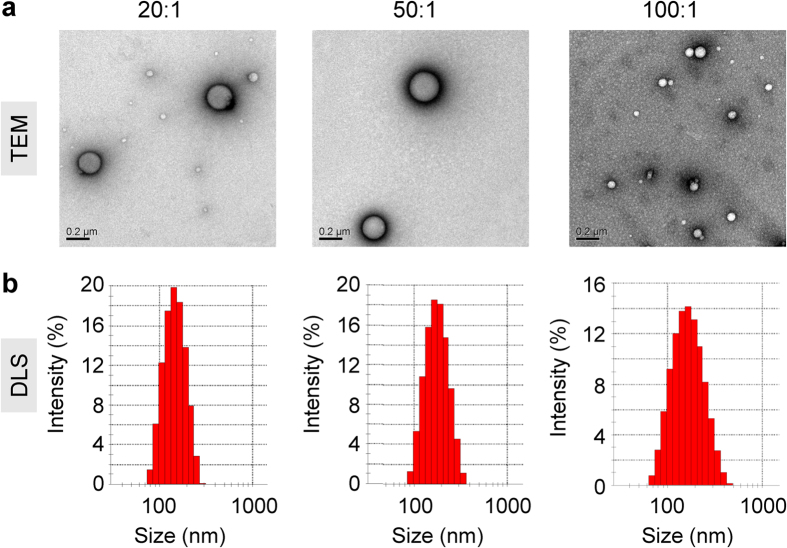
TEM images (**a**) and statistical size (**b**) of RGD10-10R/siRNA complexes with various molar ratios. The statistical sizes of the complexes were calculated according to the intensity of the DLS signal.

Transmission electron microscopy (TEM) and dynamic light scattering (DLS) were used to investigate the physicochemical properties of the complexes, including their morphology, size, polydispersity index (PDI), and zeta potential. The TEM images revealed that all three formulations, with molar ratios of 20:1, 50:1 and 100:1, exhibited regularly spherical nanostructures, with a diameter from a few tens of nanometers to three-hundred nanometers (Figs 3a and 1a). Moreover, the DLS measurements indicated that the Z-average particle sizes of the three complexes were between 110–150 nm with a PDI of less than 0.20 (Fig. 3b, Table 1). As a reference for the measurements, the DLS size of Lipofectamine 2000/siRNA complex was approximately 140 nm. As expected, the peptides alone or naked siRNAs showed no statistical size (Table 1). The zeta potentials were 5.20, 21.40, and 20.08 mV for the complexes with molar ratios of 20:1, 50:1, and 100:1, respectively. The zeta potential of the Lipofectamine 2000/siRNA complex was 24.40 mV. As expected, naked siRNA presented a negative charge of −8.38 mV (Table 1). Note that peptide/siRNA complexes with a large molar ratio (e.g., 100:1) could slowly move in the agarose gel from the positive pole to the negative pole because they presented a positive charge under such conditions (Fig. 2b-left panel).

### Cellular uptake of peptide/siRNA complexes

**Figure 4:**
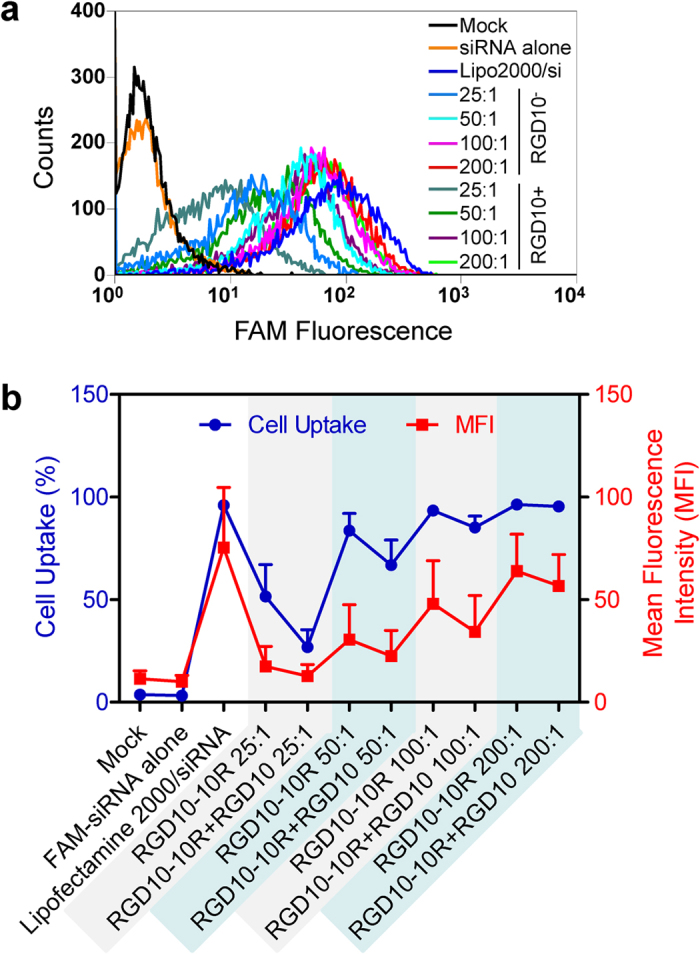
FACS-recorded internalization of the complexes by MDA-MB-231 cells. (**a**) The FAM fluorescence intensity (represents complexes taken up by cells) and the corresponding counts. (**b**) The mean fluorescence intensity (MFI) and the percentages of cellular uptake of the complexes; the red and blue lines represent the MFI and the percentage of uptake, respectively. RGD10 was used as a receptor competitor of RGD10-10R. Its addition remarkably reduced the cellular uptake of RGD10-10R complexes, indicating a mechanism of ligand/receptor interaction. Each bar represents the mean ± SEM, n = 2.

To evaluate the siRNA delivery capacity of the peptide, FACS assays were performed using the MDA-MB-231 cell line. MDA-MB-231 is a breast cancer cell line with a high expression level of integrin α_v_β_3_^35^,^36^,^37^, the receptor of the RGD10 motif. Taking advantage of this ligand/receptor interaction, drug delivery approaches targeting α_v_β_3_ have been extensively investigated^37^,^38^,^39^. As the molar ratio of peptide/siRNA increased, the percentage of the cells that internalized FAM-labeled siRNA was greatly increased (Fig. 4a,b). Accordingly, the mean fluorescence intensity (MFI) was also gradually enhanced (Fig. 4a,b). When the molar ratio reached 50:1, the peptide/siRNA complex exhibited transfection efficiency that was equal to that of Lipofectamine 2000, a gold standard for *in vitro* siRNA transfection (Fig. 4a,b). To confirm that this internalization was triggered through ligand/receptor recognition and interaction (Fig. 1b), RGD10 peptide without polyarginine at the C terminal, and thus no siRNA binding capacity, was used as a competitor of RGD10-10R for receptor binding. The results indicated that adding RGD10 reduced both the transfection efficiency and the MFI of the complexes with various molar ratios (Fig. 4b).

### Subcellular localization

**Figure 5:**
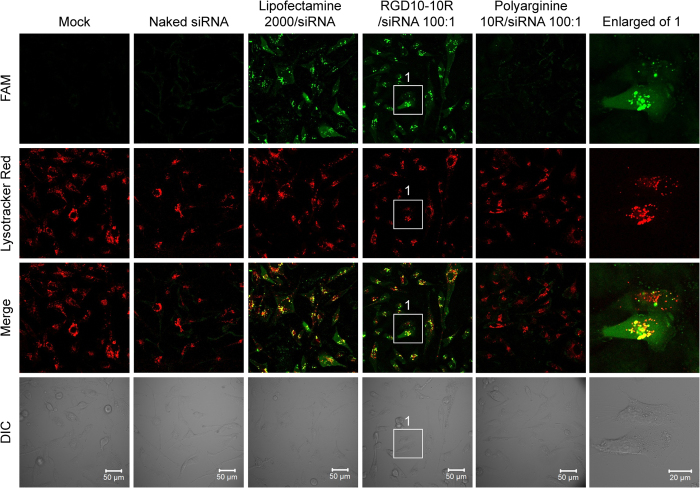
Intracellular localization of the complexes recorded by confocal microscopy (LSM510, Carl Zeiss, Germany). Confocal laser scanning microscopy (CLSM) images were acquired at 8–10 hours post-transfection. LysoTracker Red was used to stain and indicate endosomes and lysosomes. Naked siRNA was included as a negative control. The commercial transfection reagent lipofectamine 2000 and polyarginine 10R were also used as controls for this assay. siRNAs were labeled with FAM (green) fluorophore. The rightmost panel was enlarged from RGD10-10R/siRNA-treated cells that were indicated with white rectangles and number ‘1’. Scale bars (50 μm or 20 μm) are shown in the bright field images.

Confocal microscopy was used to investigate the subcellular localization of the peptide/siRNA complexes. According to the confocal microscopy observations, FAM-labeled siRNAs were internalized by the cells after being transfected for 8–10 hours, with a distribution pattern and fluorescence intensity that were similar to those of commercial Lipofectamine 2000 (Fig. 5). Magnified images of RGD10-10R/siRNA-treated cells exhibited both granular-shaped and dispersive signals. The granular signals in yellow represented the co-localization of the siRNAs (green signals) and the endosome/lysosome complexes (red signals), indicating that those siRNAs were still located in the endosomes/lysosomes. In contrast, the dispersive siRNA signals within the cytoplasm were hypothesized to be the active molecules that escaped from the endosomes/lysosome. Note that although it has been reported that polyarginine is a type of cell-penetrating peptide^40^,^41^, cells treated with only 10R/siRNA complexes showed faint siRNA signals around the cell surface (Fig. 5). This result might be attributed to the relatively weaker siRNA binding capability for 10R compared with RGD10-10R (Fig. 1b), or the peptide lost its penetrating capacity after complexing with siRNA. Similar observations were obtained for the cells treated with naked-siRNA (Fig. 5).

The FACS and confocal data (Figs 4 and 5) indicated that RGD10-10R mediated efficient siRNA transfection *in vitro* and showed that this activity was greatly compromised by the receptor competitor RGD10. Furthermore, polyarginine (10R) was unable to deliver siRNA into MDA-MB-231 cells. Collectively, siRNA transfection mediated by RGD10-10R into α_v_β_3_-highly expressed cells was a robust and specific process.

### Knockdown efficiency *in vitro*

**Figure 6:**
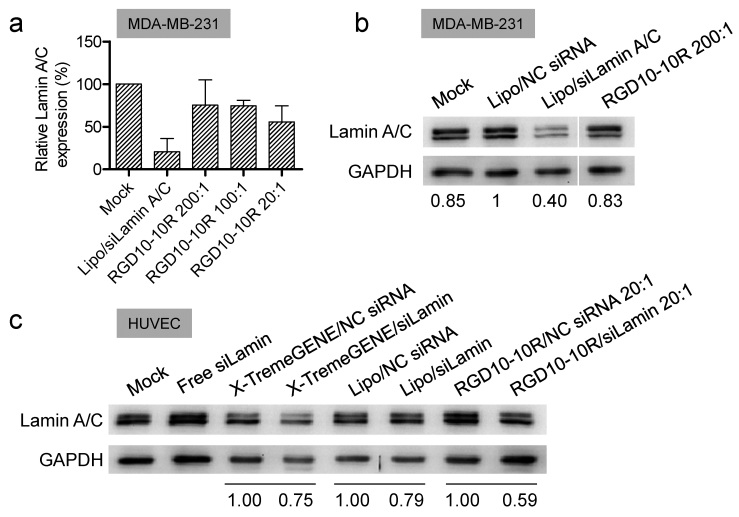
*In vitro* gene silencing efficiency verified by RT-PCR and Western blot. Relative mRNA (**a**) and protein (**b**) expression levels of Lamin A/C in MDA-MB-231 cells subjected to various treatments. (**c**) Relative protein expression of Lamin A/C examined in HUVEC, another cell that highly expresses α_v_β_3_. The commercial transfection reagents lipofectamine 2000 (Invitrogen) and X-TremeGENE (Roche) were used as controls. The numbers under the bands (**b**,**c**) represent the relative protein expression level compared to carrier/NC siRNA treated cells. Each bar represents the mean ± SD, n = 3.

Real-time quantitative PCR (RT-qPCR) and Western blot were performed to assess the *in vitro* activity of the RGD10-10R/siRNA complexes. Anti-Lamin A/C siRNA was used in this study. The RT-qPCR and Western blot data, collected from MDA-MB-231, both indicated that approximately 25% knockdown efficiency could be achieved for the complexes with high molar ratios of 100:1 and 200:1 (Fig. 6a,b). However, the complex with a lower molar ratio of 20:1 suppressed Lamin A/C gene expression with a 45% knockdown efficiency (Fig. 6a). Lipofectamine 2000/siLamin A/C, the positive control, showed a significant knockdown of targeted gene expression (~80% for mRNA, ~60% for protein) (Fig. 6a,b). In addition, human umbilical vein endothelial cell (HUVEC), another α_v_β_3_-highly expressing cell^42^,^43^, was further employed in the Western blot assay. Another commercial transfection reagent, X-TremeGENE (Roche), was included as an additional control because HUVEC is well known as being difficult to transfect. Western blot indicated that RGD10-10R/siRNA with a molar ratio of 20:1 inhibited the expression of Lamin A/C with a 41% knockdown efficiency. By contract, Lipofectamine 2000 and X-TremeGENE only exhibited 21% and 25% knockdown efficiencies, respectively (Fig. 6c). These data highlighted the pivotal role of the ligand/receptor interaction that was employed by RGD10-10R during siRNA transfection.

### Cytotoxicity

An MTT assay was conducted to investigate the biocompatibilities of the peptide/siRNA complexes in MDA-MB-231 cells. The data (Fig. S1) showed that all the formulations with molar ratios of less than 100:1 and Lipofectamine 2000/siRNA achieved nearly 100% cell viability. Complexes with a molar ratio of 200:1 and X-TremeGENE/siRNA exhibited weak and equal cytotoxicities (88% cell viability).

### *In vivo* biodistribution

**Figure 7:**
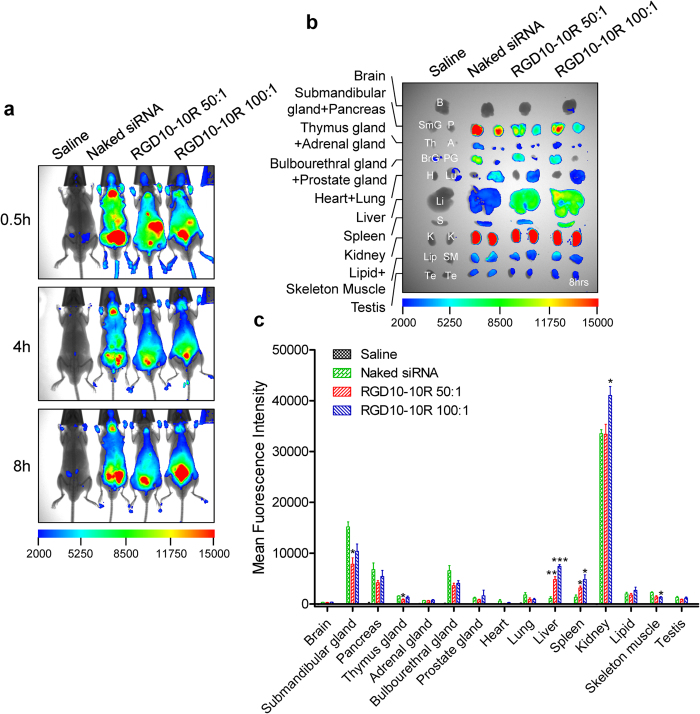
*In vivo* biodistributions of the complexes in C57BL/6 mice (2.5 mg/kg for siRNA). (**a**) Whole body imaging at given time points after administration *via* tail vein injection. (**b**) Fluorescence detection of isolated main organs of mice at the final observation time point. (**c**) Quantitative analysis of (**b**) using a molecular imaging software package (Carestream Health, USA). The data were normalized to corresponding tissues from saline-treated animals. Each bar represents the mean ± SEM of three independent experiments. *P < 0.05, **P < 0.01, and ***P < 0.001 *vs.* corresponding tissue from naked siRNA-treated mice.

*In vivo* biodistribution of RGD10-10R/siRNA complexes was performed in mice after tail vein injection. Mice were anesthetized with a gas mixture of oxygen and isoflurane to perform fluorescent imaging at indicated time points post-administration (Fig. 7). By merging the fluorescence image with an X-ray image, we found that the peptide/siRNA complexes, in contrast to naked-siRNA, remarkably accumulated in the liver and were maintained for a longer period of time. After normalizing to the MFI of saline-treated mice, the MFIs of the naked-siRNA, ‘RGD10-10R/siRNA 50:1’ and ‘RGD10-10R/siRNA 100:1’ were 1062, 4809, and 7396, respectively, in the liver 8 hours post-administration. The normalized MFIs in the spleen for the three groups were 1332, 3145, and 4795, respectively. Both naked siRNA and complex-treated mice showed strong fluorescence signals in the kidney, *via* which siRNAs would be eliminated from the body. Furthermore, mice treated with naked siRNA showed notable accumulation in several glands, including submandibular glands and the pancreas. In contrast, the mice treated with the peptide/siRNA exhibited relatively weaker signal intensity in these glands compared to the naked-siRNA. These results suggested that RGD10-10R influenced the *in vivo* biodistribution pattern of siRNAs after forming complexes.

### Tumor-targeting properties of complexes

**Figure 8:**
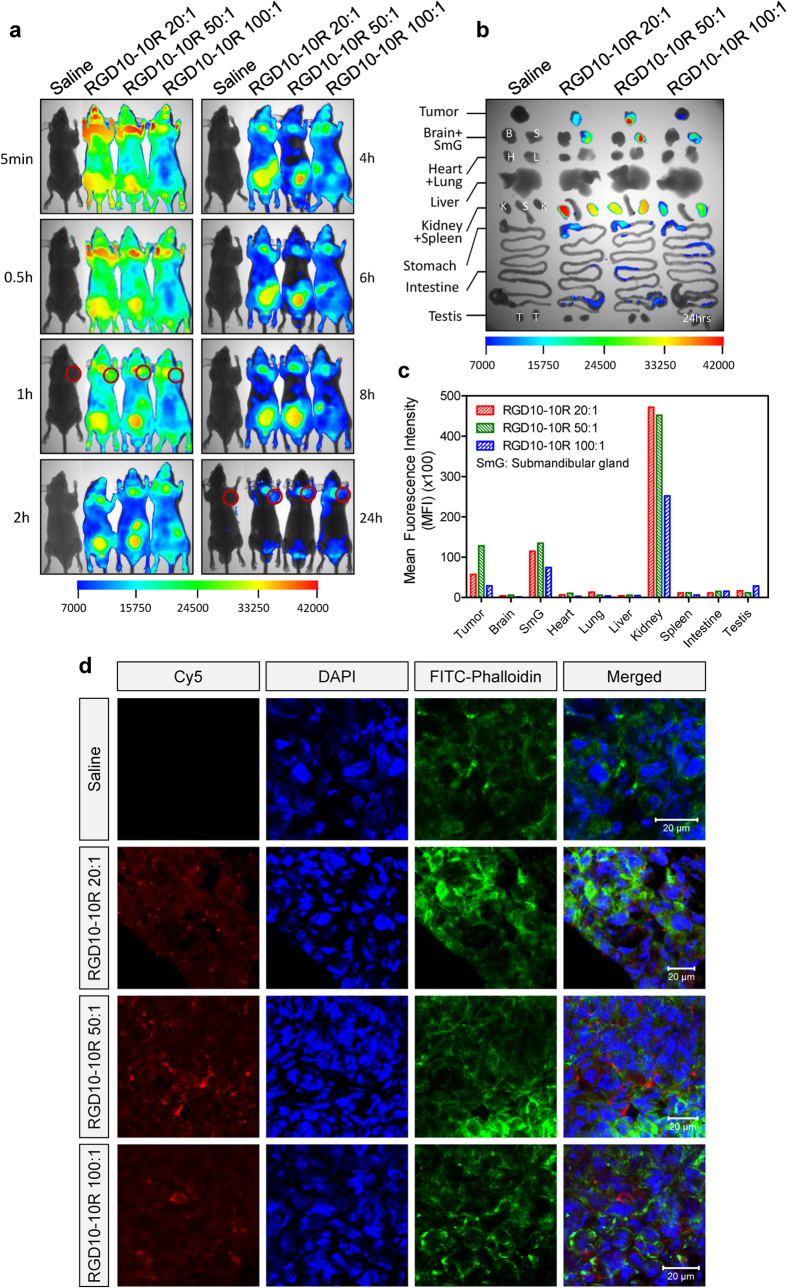
Tumor-targeted siRNA delivery mediated by RGD10-10R/siRNA complexes at 2.5 mg/kg. (**a**) Whole body imaging at given time points following intravenous injection. (**b**) Fluorescence images of siRNAs accumulated in isolated tumor tissues of BALB/c nude mice at 24 h post-injection. (**c**) Quantification of siRNAs accumulated in tumor tissues using imaging software. Data were normalized to the tumor from saline-treated animal. (**d**) Cryosections of tumor tissues observed by confocal microscopy. DAPI and fluorescein isothiocyanate-labeled phalloidin were used to stain nuclei and F actin (to show the rough cell outline), respectively. Scale bar: 20 μm.

It was previously reported that integrin α_v_β_3_ is constitutively highly expressed in tumor cells and neovascular endothelial cells^21^, suggesting its pivotal role in tumor-associated angiogenesis and potential value as a target for anti-tumor therapy. In this study, we have shown that RGD10-10R could mediate effective and specific siRNA delivery in MDA-MB-231 and HUVEC (Figs 4, 5, 6) *in vitro*. Therefore, *in vivo* tumor-targeting properties were investigated using the MDA-MB-231 xenograft murine model. Tumor cells were injected subcutaneously into the right axillary fossa of female BALB/C nude mice (Fig. 8a, as indicated by kermesinus circles). The data demonstrated that peptide/siRNA complexes remarkably accumulated in the tumor after i.v. injection (Fig. 8a). Isolated-organ-imaging at 24 hours post-administration indicated that RGD10-10R/siRNA with a molar ratio of 50:1 exhibited the highest fluorescence intensity compared with those with molar ratios of 20:1 and 100:1. For clarification, the absolute MFIs in Figs 7 and 8 are not comparable because these two assays were performed under different experimental conditions.

Furthermore, cryosections were prepared and stained with DAPI and FITC-labeled phalloidin. Confocal scanning microscopy revealed that the red signals, representing Cy5-labeled siRNAs, were localized between the blue and green areas, representing nuclei and cell outlines, respectively (Fig. 8d). Therefore, it is highly likely that siRNAs were delivered into cytoplasm of the tumor cells, where RNA interference (RNAi) occurs.

## Discussion

The development of siRNA therapeutics is severely hindered by inadequate clinically suitable, safe and effective drug delivery vehicles. In this work, we designed a bifunctional peptide, RGD10-10R. The RGD10 motif is the specific ligand of integrin α_v_β_3_, an integrin that is highly expressed in tumor cells and neovascular endothelial cells, and decu-L-arginine (10R) can bind siRNA through electrostatic interactions.

It was demonstrated that RGD10-10R can effectively form regular nanostructures with siRNA, and it protected siRNA from RNase attack. We observed that RGD10-10R/siRNA complexes might run in the opposite direction (from the positive pole to the negative pole) when the molar ratio increased to more than 100:1 (Fig. 2b-left panel), suggesting that the net charge of the complexes with this molar ratio was converted from negative to positive. Polyarginine (10R) without the RGD10 polypeptide at the N terminal showed a weaker siRNA binding capacity than RGD10-10R, which might be because the longer peptide chain was beneficial for siRNA complexation and complex stabilization. In addition, the RNase resistance assay performed with unstable RNA, mmu-miR-672, should be attributed to the steric hindrance present within the peptide/siRNA complexes.

RGD10, as a receptor competitor of RGD10-10R, was introduced into the cellular uptake assay to validate the transfection mechanism of RGD10-10R. The addition of RGD10 remarkably reduced the cellular uptake of RGD10-10R/siRNA, suggesting that the transfection of siRNAs into the cell was indeed dependent on the ligand/receptor interaction. Furthermore, RT-PCR and Western blot assays revealed that anti-Lamin A/C siRNA repressed target gene expression *in vitro* after being transfected with RGD10-10R. Lamin A/C, also known as LMNA, is a component of the nuclear membrane structure that, in humans, is encoded by the LMNA gene^44^. Lamins are important for maintaining normal cell functions, such as cell cycle control, DNA replication and chromatin organization. During apoptosis, Lamin A/C is specifically cleaved to a large (40–45kD) and a small (28kD) fragment. The cleavage of lamins results in nuclear dysregulation and cell death^45^. Dysfunction of Lamin A/C is associated with a series of diseases^46^. However, the *in vitro* activity of RGD10-10R/siRNA was compromised, which might result from insufficient particles escaping from the endosome/lysosome after being internalized by the cells. This was supported by the subcellular localization data (Fig. 5, yellow granule). It is also possible that the binding force between the peptides and the siRNAs is too strong to release siRNA, even though the complexes had successfully escaped from the endosomes/lysosomes.

The *in vivo* distribution profile revealed that the complexes not only remarkably accumulated in the liver in normal C57BL/6 mice but could also effectively deliver siRNA into tumor tissue in the xenograft murine model. The size distribution of the peptide/siRNA complexes ranged from 110–150 nm (Fig. 1b, Table 1). It was previously reported that the particle size should be less than 200 nm if it is intended to deliver drugs to hepatocytes because larger particles are more likely processed and eliminated by Kupffer cells and filtered into the spleen, for which the cut-off point extends up to 250 nm^47^. However, it was also reported that fenestrations in the liver sinusoidal endothelium permitted particles of 100–200 nm in diameter to exit the bloodstream and gain access to hepatocytes and other liver cells^48^,^49^, which might facilitate the accumulation of siRNAs or particles in the liver. In addition, this size distribution pattern also met the requirements of the EPR (enhanced permeability and retention) effect, which facilitates the accumulation of nanoparticles in tumor tissue in a passive-targeting manner^50^. Together with the scenario that integrin was highly expressed in both MDA-MB-231 and neovascular endothelial cells, RGD10-10R/siRNA could be entrapped in the tumor tissue and cross the cell membrane *via* receptor-mediated endocytosis. Furthermore, the *in vivo* imaging data revealed that RGD10-10R/siRNA with a molar ratio of 50:1 showed more tumor accumulation than the complexes with molar ratios of 20:1 and 100:1. The DLS size of ‘RGD10-10R/siRNA 50:1′ was 117 nm, the smallest particle size among the three formulations (Table 1), indicating that particle size indeed played an important role in facilitating tumor-targeted transport of siRNA^50^.

In conclusion, RGD10-10R provided a promising siRNA delivery tool for further development of RNAi-based therapeutics, particularly for cancer treatment.

## Materials and methods

### Materials

RGD10-10R, with an amino acid sequence of DGARYCRGDCFDGRRRRRRRRRR, a molecular formula of C_117_H_201_N_59_O_31_S_2_, and a molecular weight of 2994.41 g/mol, was synthesized by Invitrogen (USA). The purity as determined by HPLC was 91.19%. RGD10, a receptor-binding competitor of RGD10-10R, and polyarginine (10R) were purchased from Beijing SciLight Biotechnology Ltd. Co. (Beijing, China). The reverse-transcription kit and all reagents used for real-time PCR were supplied by TIANGEN Biotech (Beijing) Co., Ltd. (Beijing, China). SYBR Green was obtained from Invitrogen (USA). Lamin A/C monoantibody and horseradish peroxidase (HRP)-labeled goat anti-rabbit secondary antibody were purchased from Cell Signaling Technology, Inc. (Danvers, MA, USA) and Bio-Rad Laboratories (Hercules, CA, USA), respectively. GAPDH antibody and DAPI (4′,6-diamidino-2- phenylindole) were provided by Zhongshan Goldenbridge Biotechnology Co. Ltd. (Beijing, China). The transfection regent Lipofectamine^TM^ 2000 and X-TremeGENE were obtained from Invitrogen (USA) and Roche (Basel, Switzerland), respectively. FAM-labeled NC siRNA, siLamin A/C, si-NC67-M and mmu-miR-672 were provided by Shanghai GenePharma Co, Ltd. (Shanghai, China). Cy5-labeled NC siRNA was supplied by Suzhou Ribo Life Science Co., LTD. (Kunshan, China). The sequences were as follows: FAM-labeled NC siRNA: sense strand: 5′-FAM-CCUUGAGGCAUACUUCAAAdTdT-3′, the fluorophore FAM was labeled at 5′ of sense strand; antisense strand: 5′-UUUGAAGUAUGCCUCAAGGdTdT- 3′. siLamin A/C: sense strand: 5′-CUGGACUUCCAGAAGAACAdTdT-3′; antisense strand: 5′-UGUUCUUCUGGAAGUCCAGdTdT- 3′. si-NC67-M: sense strand: 5′-UCACAACCUCCUAGAAAGAGUAGA-3′; antisense strand: 5′-UACUCUUUCUAGGAGGUUGUUAUU-3′. mmu-miR-672: sense strand: 5′-UGAGGUUGGUGUACUGUGUGUGA-3′; antisense strand: 5′-ACACACAGUACACCAACCUUAUU-3′. Cy5-labeled NC siRNA: sense strand: 5′-Cy5-CCUUGAGGCAUACUUCAAAdTdT-3′, the fluorophore Cy5 was also labeled at 5′ of the sense strand; antisense strand: 5′-UUUGAAGUAUGCCUCAAGGdTdT- 3′. Moreover, to stabilize the sequence and provide it with the ability to resist RNase attack, several bases of Cy5-NC siRNA were modified with 2′-Ome or fluoro.

### RGD10-10R/siRNA complex preparation

According to the requirements of the present experiment, RGD10-10R powder was dissolved in 200–1000 μM (approximately 0.60–3.0 μg/μl) (for *in vitro* testing) or 10–30 μg/μl (for *in vivo* study) with DEPC (diethylpyrocarbonate)-treated water. Moreover, siRNA was dissolved in 20 μM (approximately 0.266 μg/μl) and 1 μg/μl with DEPC water for *in vitro* tests and *in vivo* studies, respectively. The peptide and siRNA solutions were mixed directly according to different desired molar ratios. After electrostatic interaction for 15–20 min at room temperature, peptide/siRNA complexes were formed (Fig. 1a). Additional DEPC water or 1 × PBS or normal saline (NS) could be added to the complex solution to obtain the final formulation used for transfection or injection.

### Gel retardation assay

To assess the siRNA loading ability of RGD10-10R, a gel retardation assay was performed. Here, a serum-stable siRNA, si-NC67-M^51^, was used to prepare the peptide/siRNA complexes according to the aforementioned protocol. Twenty-two and a half microliters of complexes containing 50 pmol siRNA (20 μM, 2.5 μl) was mixed with 4 μl of 6 × loading buffer and loaded into a 4 wt% agarose gel containing 5 μg/ml ethidium bromide. Electrophoresis was set up in 1 × TAE buffer at 120 V and run for 40 min. siRNA retardation was analyzed on a UV illuminator to show the location of the siRNA.

### RNase resistance

Theoretically, peptides can protect siRNAs form RNase attack because of the steric hindrance effect. Therefore, siRNAs entrapped in the complexes would be more stable than naked siRNAs. Hence, an RNase resistance assay was performed. mmu-miR-672, a microRNA that was previously proven to be unstable in fetal bovine serum (FBS)^52^, was employed in this assay. Chemically synthesized microRNA is similar to siRNA in terms of their nucleotide base constituents (cytosine, guanine, adenine and uracil) and biological structure properties (double strand, 19–25nt). A RGD10-10R/RNA complex with a molar ratio of 100:1 and containing 22.5 pmol of mmu-miR-672 was prepared. Additionally, a 22.5 pmol naked mmu-miR-672 solution without peptide binding was used as a control. Peptide/RNA complexes and naked mmu-miR-672 were incubated in 10% FBS (v/v, in 1 × PBS) at 37 °C. Complexes and naked RNA were collected and immediately frozen at −20 °C at 0 h, 3 h, 6 h and 12 h post-incubation. Then, all samples were centrifuged for 5 min at 10000 rpm and 4 °C to precipitate complexes or naked RNA. The supernatants were discarded, and 15 μl of DEPC water was added to resuspend the precipitate. To destroy complexes and release RNA, proteinase K (0.2 μl, 1 mg/ml), Tris-HCl (1 μl, 50 mM, pH = 7.0) and CaCl_2_ (1 μl, 5 mM) were added to the resuspended complex solution, followed by incubating for 4–5 hours at 37 °C. After adding loading buffer, the complexes and naked RNA solution were both separated in native 20% PAGE (polyacrylamide gel electrophoresis) for 150 min at a constant voltage of 150 V. Finally, the gels were stained with SYBR Gold for 20 min, exposed by Vilber Lourmat imaging system (France) and analyzed with Biocap software to show the location of the small RNA.

### Characterization of particle sizes and zeta potential

The particle sizes and zeta potentials of the peptide/siRNA complexes were measured using a Zetasizer 3000HS (Malvern Instruments, Inc., Worcestershire, UK) at a wavelength of 677 nm with a constant angle of 90° at room temperature. Complexes were prepared by mixing 2 μg of si-NC67-M (7.69 μl, 20 μM) and the required amount of peptide (1000 μM) at room temperature. The solutions were then diluted with 0.8 ml of double-distilled water prior to characterization. Moreover, the morphologies and sizes of the complexes were further analyzed by transmission electron microscopy (TEM) (Tecnai G2 20 STWIN transmission electron microscope, Philips, Netherlands) with an acceleration voltage of 200 kV. Briefly, samples were prepared by adding 7 μl of complex solutions onto a carbon-coated copper grid. Then, they were negatively stained with 1% (wt/v) phosphotungstic acid (pH adjusted to 7.3 with 1N NaOH) and air-dried prior to collecting images.

### Cell lines and culture

The breast carcinoma cell line MDA-MB-231 was obtained from the Cell Resource Center of Peking Union Medical College (Beijing, China). Cells were cultured with L15 medium supplemented with 10% fetal bovine serum, 100 units/ml penicillin and 100 μg/ml streptomycin at 37 °C in a 100% air humidified atmosphere (without CO_2_). CO_2_ was isolated from the cells by covering the culture plates or bottles with Parafilm. Human umbilical vein endothelial cells (HUVECs) were grown in medium 199 (Invitrogen) containing fibroblast growth factor, heparin and 20% fetal bovine serum (HyClone, Ogden, UT) at 37 °C in a humidified atmosphere of 5% CO_2_.

### Fluorescence-activated cell sorting (FACS)

MDA-MB-231 cells were plated in 12-well plates (1 × 10^5^ per well) one day before transfection and incubated in L15 medium supplemented with 10% FBS, 100 U/ml penicillin, and 100 mg/ml streptomycin to 60–70% confluence. Then, the medium was removed and replaced with Opti-MEM, a reduced serum medium designed for cationic lipid transfections. Subsequently, complexes containing 100 pmol of FAM-labeled siRNA were added into each well. After incubation at 37 °C for 4 hours, 2 ml of fresh L15 containing 10% FBS was added, followed by further incubation for 4 hours. Then, the cells were washed three times with 1 ml of precooled 1 × PBS to remove residual free complexes and siRNAs, subsequently suspended in 400 μl of precooled 1 × PBS and introduced into a FACSCalibur flow cytometer (Becton Dickinson, San Jose, CA, USA).

### Subcellular localization

MDA-MB-231 cells were plated in 6-well plates (2 × 10^5^ per well) with glass coverslips (one coverslip per well) at the bottom one day prior to transfection. RGD10-10R/siRNA complexes containing 150 pmol (7.5 μl, 20 μM) of FAM-labeled siRNA were transfected into cells according to the above protocol. Approximately 10 hours later, the subcellular distribution of siRNA was observed in living cells using a Zeiss confocal microscope (LSM510, Carl Zeiss, Germany). Moreover, LysoTracker Red DND-99 (Invitrogen, Carlsbad, CA) was used to indicate endosomes and lysosomes.

### MTT assay

An MTT assay was employed to evaluate the cytotoxicity of the peptide/siRNA complexes. MDA-MB-231 cells were seeded at 10000 cells per 100 μl of L15 per well on a 96-well plate and allowed to adhere overnight at 37 °C. Cells were transfected with complexes containing 6.25 pmol of siRNA in 25 μl of Opti-MEM. Four hours later, 200 μl of fresh complete L15 medium was added. After an additional 20 hours of culture in the presence of 10% FBS, 200 μl of medium was removed and 2 μl of MTT was added to each well, followed by an additional 4 hours of incubation under the same incubation conditions. Then, all medium was removed and 50 μl of DMSO was added, followed by incubation for an additional 30 min at 37 °C to dissolve formazan. Finally, the absorbance was read at 540 nm with a reference wavelength of 650 nm, and the absolute absorbance (OD_net540_) was OD_540_ minus OD_650_. For comparison of relative viability, all data were presented as the mean percentage ± S.D. in two replicate samples compared to the absorbance value of mock-treated cells. The cell viability was calculated as follows:


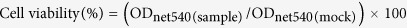


where OD_net540(sample)_ was the absorbance at 540 nm of the transfected cells and OD_net540(control)_ was the absorbance at 540 nm of the mock control (nontransfected cells).

### Real-time PCR

Real-time PCR was performed to determine whether siLamin A/C (against the LMNA gene) transfected by RGD10-10R could suppress the expression of the targeted gene. MDA-MB-1231 cells were plated in 12-well plates (1 × 10^5^ per well) one day prior to transfection and incubated in L15 to approximately 60% confluence. Then, the medium was removed and replaced with 1 ml of Opti-MEM. Subsequently, 200 μl of complexes containing 100 pmol siLamin A/C was added to each well (the final transfection concentration of siRNA was ~80 nM). Here, Lipofectamine 2000 was the positive control, and each sample was tested in two replicate wells. After 4 hours of incubation, 2 ml of fresh L15 containing 10% FBS was added, and the cells were further incubated for 20 hours. The total RNA of cells was extracted with TRIzol according to the manufacturer’s protocol. Cells from the two replicates were mixed prior to RNA extraction. Then, cDNA was prepared by two-step reverse transcription. First, 2 μg of total RNA, 1 μl of oligo dT and ddH_2_O (total of 5 μl) was mixed and incubated for 5 min at 70 °C, followed by transferring onto ice immediately. Then, 5.5 μl of RNase-free water, 4 μl of 5 × reaction buffer, 2.5 μl of MgCl_2_ (500 mM), 1 μl of dNTP (10 mM), 1 μl of RNase inhibitor, and 1 μl of reverse-transcription enzyme were added to the former precooled tube, followed by reaction at 25 °C for 5 min, 42 °C for 1 hour and 75 °C for 15 min. A real-time PCR reaction system (0.1 μl of Tag enzyme, 0.5 μl of dNTP (10 mM), 2.5 μl of 10 × reaction buffer, 1 μl of SYBR Green, 0.75 μl of MgCl_2_ (500 mM), 18.15 μl of RNase-free water, 1 μl of cDNA, and 1 μl of primer mixture (20 μM)) was prepared and first hot-started for 5 min at 95 °C before 40 cycles of 30 sec at 95 °C, 30 sec at 55 °C and 30 sec at 72 °C. After the melting procedure completed, the samples were stored at 4 °C. The expression level of Lamin A/C was analyzed by the Ct (cycle threshold) values using a standard protocol.

### Western blot

MDA-MB-231 cells or HUVECs were seeded in 6-well plates (2 × 10^5^ per well). siRNA transfection was then completed using complexes containing 100 pmol of siLamin A/C. After incubation for 48 hours, total protein was extracted using a standard protocol. The protein-extracted lysate was resuspended in 75 μl of protein lysis buffer containing 1 μl of proteinase inhibitor cocktail. Western blots were conducted using a wet system at a loading of 20 μg of protein. Blots were incubated overnight at 4 °C with the monoclonal antibody diluted to 1:1000 (for lamin A/C) or 1:5000 (for GAPDH), followed by incubation with HRP-conjugated goat anti-rabbit (for lamin A/C) or anti-mouse (for GAPDH) secondary antibodies (1:5000; Zhongshan Goldenbridge Biotechnology Co. Ltd., Beijing, China) at room temperature for 2 hours. Finally, the membranes were exposed using Bio-Rad Universal Hood II (Bio-Rad, Bossier City, LA).

### Animals

Male C57BL/6 mice (for *in vivo* distribution assay, weighing 18–22 g) and female BALB/c nude mice (for tumor-targeting assessment, weighing 18–22 g) were purchased from the Academy of Military Medical Sciences of China. The animals were maintained in the Peking University Laboratory Animal Center (an AAALAC-accredited and specific-pathogen-free (SPF) experimental animal facility). All procedures involving experimental animals were performed in accordance with protocols approved by the Institutional Animal Care and Use Committee (IACUC) of Peking University.

### Biodistribution of complexes in C57BL/6

To evaluate the biodistribution properties of RGD10-10R/siRNA complexes, male C57BL/6 mice 5–7 weeks old, weighing 18–22 g, were used to perform *in vivo* imaging. The mouse abdomens were shaved one day prior to administration to facilitate observation. A given formulation was administered to each mouse *via* tail vein injection at a dose of 2.5 mg/kg siRNA. The Cyanine Cy5 fluorescence signal of the whole body was examined using a Kodak *in vivo* imaging system (Kodak In-Vivo Imaging System FX Pro, Carestream Health, USA) at given time points. Mice were anesthetized with a gas mixture of oxygen and isoflurane using a Matrix VIP3000 Isoflurane Vaporizer (Matrix, USA) during imaging. At the end-point, mice were sacrificed by cervical dislocation, and the major organs were isolated and examined. The above experiments were replicated three times independently. Quantitative analyses were performed using a molecular imaging software package (Carestream Health, USA).

### *In vivo* tumor-targeted delivery and cryosection assay

To validate the tumor-targeting property of RGD10-10R, 5 × 10^6^ MDA-MB-231 cells were subcutaneously injected into the right axillary fossa of female BALB/C nude mice. When the tumors had grown to approximately 400 mm^3^, the indicated formulations were intravenously administered to each mouse. The mice were anesthetized with an intraperitoneal (i.p.) injection of pentobarbital sodium (50 mg/kg), and images were acquired at indicated time points using a Kodak *in vivo* imaging system. Twenty-four hours later, the mice were sacrificed by cervical dislocation, and the major tissues including tumors were isolated and re-examined. Then, the tumors were placed in OmniSette tissue cassettes, embedded in OCT, and frozen in a pre-chilled Dewar flask containing a liquid nitrogen/dry ice slurry for ~1 min until the OCT turned white and opaque. Subsequently, the specimens were cut into 10 μm sections on a cryostat. The sections were stained by DAPI to visualize the nucleus and FITC-labeled phalloidin to visualize the F actin. The rough cell outline was revealed by F actin. Finally, the cryosections were observed using a confocal microscope (LSM510, Carl Zeiss, Germany).

### Statistical analysis

Data were expressed as the mean ± SD or as the mean ± SEM, as indicated in the figure legends. Statistical variance was calculated by *t*-test, and P < 0.05 was considered statistically significant.

## Additional Information

**How to cite this article**: Huang, Y. *et al.* Systemic Administration of siRNA *via* cRGD-containing Peptide. *Sci. Rep.* **5**, 12458; doi: 10.1038/srep12458 (2015).

## Supplementary Material

Supplementary Information

## Figures and Tables

**Table 1:** Physicochemical characteristic of RGD10-10R/siRNA complexes with various molar ratios.

**Formulation**	**Size (nm)**	**PDI**	**ZP (mV)**	**Mob (μmcm/Vs)**	**Cond (mS/cm)**
RGD10-10R/siRNA 20:1	152.02 ± 77.27	0.115 ± 0.068	5.20 ± 1.49	0.408 ± 0.117	0.0546 ± 0.0176
RGD10-10R/siRNA 50:1	116.86 ± 61.90	0.150 ± 0.083	21.40 ± 7.92	1.677 ± 0.621	0.0294 ± 0.0033
RGD10-10R/siRNA 100:1	144.51 ± 39.82	0.172 ± 0.034	20.08 ± 2.69	1.575 ± 0.211	0.0465 ± 0.0063
Lipofectamine 2000/siRNA	140.5 ± 15.27	0.261 ± 0.040	24.40 ± 7.01	1.913 ± 0.548	0.0498 ± 0.0271
Naked siRNA	ND	ND	−8.38 ± 2.54	−0.657 ± 0.199	0.0418 ± 0.0232

The data are shown as the mean ± SD, n = 4–6.

Abbreviations: PDI: polydispersity index; ZP: zeta potential; Mob: electrophoretic mobility; Cond: conductivity; and ND: not detectable
