# Supplementary material for: Systemic Administration of siRNA via cRGD-containing Peptide
Source: Sci Rep. 2015 Aug 24;5:12458. doi: 10.1038/srep12458 (PMC4547141; doi:10.1038/srep12458)
Supplement: Supplementary Information [file srep12458-s1.pdf]

Supplementary Material

Systemic Administration of siRNA *via* cRGD-containing Peptide

Yuanyu Huang[1*], Xiaoxia Wang[1], Weiyan Huang[1], Qiang Cheng[1], Shuquan Zheng[1], Shutao Guo[2], Huiqing Cao[1], Xing-Jie Liang[2], Quan Du[1*], Zicai Liang[1,3*]

[1] Institute of Molecular Medicine; State Key Laboratory of Natural and Biomimetic Drugs, School of Pharmaceutical Sciences, Peking University, Beijing 100871, China

[2] Chinese Academy of Sciences Key Laboratory for Biomedical Effects of Nanomaterials and Nanosafety, National Center for Nanoscience and Technology of China, Beijing 100190, China

[3] Collaborative Innovation Center of Chemical Science and Engineering (Tianjin), Tianjin 300072, China

[*] Correspondence should be addressed to Z. Liang (liangz@pku.edu.cn, Tel./fax: +86-10-62769862) or Q. Du (quan.du@pku.edu.cn, Tel./Fax: +86-10-82805780) or Y. Huang (yyhuang@pku.edu.cn, Tel: +86-10-62750683)

Supplementary Figure S1

Cytotoxicity of RGD10-10R/siRNA complexes

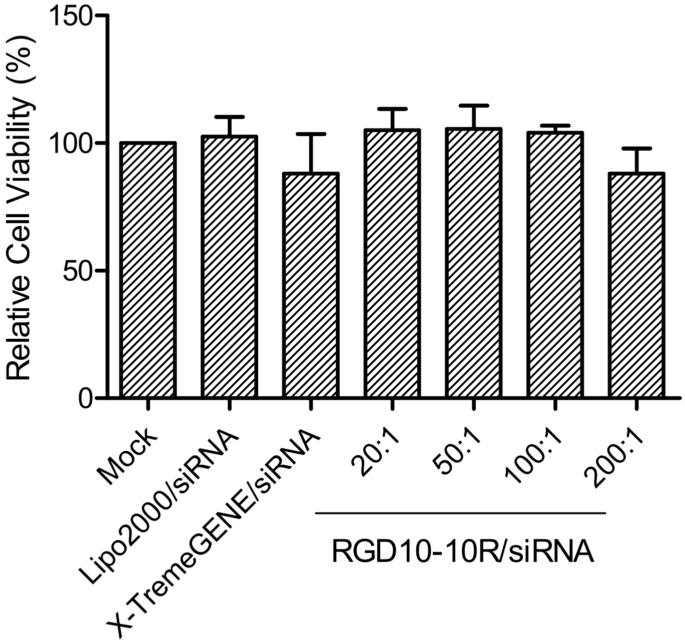

Fig. S1. Relative cell viability of MDA-MB-231 treated with various formulations. Each bar represents the mean ± SD, n=2.
